# The Impact of Environmental Heterogeneity and Life Stage on the Hindgut Microbiota of *Holotrichia parallela* Larvae (*Coleoptera: Scarabaeidae*)

**DOI:** 10.1371/journal.pone.0057169

**Published:** 2013-02-21

**Authors:** Shengwei Huang, Hongyu Zhang

**Affiliations:** State Key Laboratory of Agricultural Microbiology, Institute of Urban and Horticultural Pests, and Hubei Insect Resources Utilization and Sustainable Pest Management Key Laboratory, College of Plant Science and Technology, Huazhong Agricultural University, Wuhan, China; University of Waterloo, Canada

## Abstract

Gut microbiota has diverse ecological and evolutionary effects on its hosts. However, the ways in which it responds to environmental heterogeneity and host physiology remain poorly understood. To this end, we surveyed intestinal microbiota of *Holotrichia parallela* larvae at different instars and from different geographic regions. Bacterial 16S rRNA gene clone libraries were constructed and clones were subsequently screened by DGGE and sequenced. *Firmicutes* and *Proteobacteria* were the major phyla, and bacteria belonging to *Ruminococcaceae*, *Lachnospiraceae*, *Enterobacteriaceae*, *Desulfovibrionaceae* and *Rhodocyclaceae* families were commonly found in all natural populations. However, bacterial diversity (Chao1 and Shannon indices) and community structure varied across host populations, and the observed variation can be explained by soil pH, organic carbon and total nitrogen, and the climate factors (e.g., mean annual temperature) of the locations where the populations were sampled. Furthermore, increases in the species richness and diversity of gut microbiota were observed during larval growth. *Bacteroidetes* comprised the dominant group in the first instar; however, *Firmicutes* composed the majority of the hindgut microbiota during the second and third instars. Our results suggest that the gut's bacterial community changes in response to environmental heterogeneity and host's physiology, possibly to meet the host's ecological needs or physiological demands.

## Introduction

It is well known that a wide diversity of insects harbor a number of commensal and mutualistic micro-organisms that have important ecological and evolutionary effects on their hosts [1], [2], [3], [4]. Previous studies have shown that these microbial communities can provide their hosts with many rewards, including a supply of nutrients [1], [4], [5], [6], [7], [8], contributions to host reproduction and survival [9], [10], protection against natural predators and parasites [11], [12], detoxification [13], [14], and enhanced social interactions [15]. Although the abundance and distribution of these microbial communities have been recently explored in diverse insects, how environmental factors and host physiology affect the composition and diversity of the intestinal microbiota in the host is mostly unknown [10], [16], [17]. It has been shown that physicochemical parameters can control the vertical distribution of marine bacterial communities [18], [19]. Furthermore, climate change [20], soil attributes [21], and plant species [22] are also important in determining the structure and composition of soil bacterial communities. However, whether and how the environmental conditions influence the structure of the bacterial communities in the guts of soil-dwelling insects remains poorly understood.

Scarab larvae live in soil and feed on plant roots and other organic matter of low nutritive value [23]. Like the bulbous paunch of termites, the hindgut of scarab larvae expands to a structure called the fermentation chamber, which houses dense microbial communities [24], [25], [26], [27], [28]. Additionally, previous studies have revealed that large and morphologically diverse microbial communities, as well as numerous novel lineages, are typically found in the intestinal tract of scarab larvae [27], [29]. These characteristics suggest that scarab larvae could serve as a useful model to study the relationships between the gut's microbial community and the host. However, scarab larvae are widely distributed around the globe, and the larval feeding ability differs significantly between the first instar and the second and third instars. It is believed that the scarab's dense gut bacteria may contribute to digestion, nutrition and methanogenesis [24], [30], [31], but questions remain about how ecological factors and the host's developmental stage affect the bacterial community in the gut of the scarab larvae.

The large black chafer *Holotrichia parallela* is an important peanut pest in China, and it causes great economic losses [32]. The larvae of *H. parallela* live in the soil and prefer to feed on peanuts. They also have an enlarged bulbous hindgut like other scarab beetles. In the present study, the hindgut bacterial community in *H. parallela* larvae from different geographic locations and instars were investigated using 16S rRNA clone library construction in combination with Denaturing Gradient Gel Electrophoresis (DGGE). By surveying the bacterial composition and variation of these communities, we examined the following subjects: (i) the composition and diversity of gut bacterial communities of *H. parallela* larvae; (ii) whether the community composition differs between natural population or larval stages; and (iii) if variation exists, whether and how environmental factors or life stages account for this variation. This information is important for the understanding of the symbiotic relationship between the bacteria and the scarab, as well as the mechanisms that determine gut microbiota composition and dynamics.

## Materials and Methods

### Ethics Statement

*H. parallela* has not been notified under any act or laws and rules thereof of the Government of China or any of the Province governments of Liaoning, Tianjin, Shandong, Hubei, Zhejiang, Fujian, Guangdong, Guangxi, Sichuan, Ningxia as an endangered or threatened species restricting or regulating its collection and observation. No permits were required, for collecting the larvae from the field since all the land accessed is not privately-owned or protected in any way, and *H. parallela* is not an endangered species affecting the biodiversity status.

### Insect rearing and collection

In this study, all animals were handled in strict accordance with good animal practice as defined by the relevant national and/or local animal welfare bodies. A laboratory population of *H. parallela* was reared in a terrarium filled with organic soil held at 27±1°C and on a 14:10 h light-dark photoperiod. Different instar larvae were fed with potatoes on the soil surface. Before dissection, individual larvae were selected and maintained separately in soil-free sterile containers and kept from food for 3 days, to reduce food residue in the gut; however, absorbent cotton soaked with sterile distilled water was placed into the containers and replaced every day.

**Figure 1:**
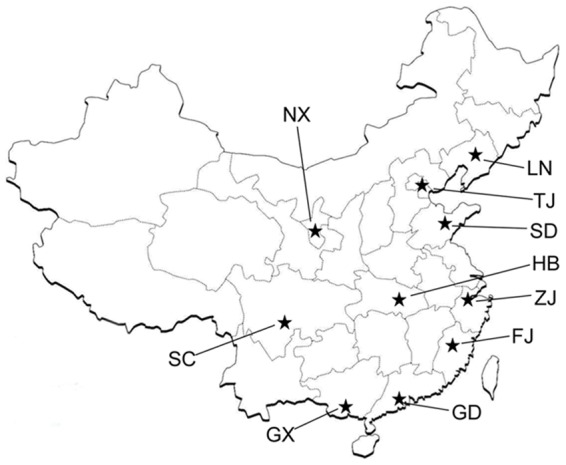
Collection sites of *H. parallela* larvae. The abbreviations of the province or municipality used are as follows: LN, Liaoning; TJ, Tianjin; SD, Shandong, HB, Hubei; ZJ, Zhejiang; FJ, Fujian; GD, Guangdong; GX,Guangxi; SC, Sichuan; NX, Ningxia. In this study, the ten natural populations of the *H. parallela* were named according to the abbreviation of the province or municipality where they were collected (for example, if the larvae were collected at Hubei province, the population was named HB).

Third-instar larvae of *H. parallela* were field-collected from ten different locations throughout China (Table 1, Fig. 1) from July through September 2008. These sampling regions were selected due to their different ecoclimatic characteristics and because the white grubs of *H. parallela* cause significant damage to the peanut production of these areas. From each region, 20–30 larvae were caught. After returning to the laboratory from the collection sites, all of the larvae were maintained separately in soil-free sterile containers and dissected immediately. Soil samples from the immediate surroundings of the larvae at each site were also collected for analysis.

**Table 1:** Characteristics of 10 collection sites and 16S rRNA gene sequence clone libraries and species richness estimates.

Population name a	Site description^b^	N^c^	N^d^	No. of OTUs^e^	Chao1	Simpson	Shannon(*H′*)	Coverage^f^
SC	peanut	166	55	43	58.3	18.58	3.22	87.9%
NX	spring wheat	165	49	40	57.2	19.24	3.20	90.3%
GX	peanut	163	54	47	62.1	25.73	3.45	88.3%
GD	peanut	168	78	49	66.1	31.73	3.54	87.5%
HB	peanut	168	60	36	52.5	20.65	3.18	92.3%
TJ	peanut	165	44	35	38.8	25.06	3.29	95.6%
FJ	peanut	165	63	48	86.5	26.84	3.45	86.1%
SD	peanut	166	68	41	48.8	21.84	3.28	92.2%
ZJ	peanut	167	78	49	60.8	27.07	3.49	88.6%
LN	corn	161	45	41	49.8	24.39	3.36	91.3%

a Population was named according to the abbreviation of the province or municipality where they were collected.

b Crop grown at the field where *Holotrichia parallela* larvae were collected.

c Number of clones isolated from the host population.

d Number of representative clones that were chosen and sequenced.

e Number of OTUs identified in the 16S rRNA gene clone library. OTUs were defined based on 3% sequence divergence.

f library coverage calculated by the equation C = (1−n/N)*100, where n is the number of unique clones and N is the total number of clones examined.

### Insect dissection

Individual healthy larvae were dissected according to the modified method described by Zhang and Jackson [28]. The intestinal tract was carefully removed from the body; the hindgut (average weight 0.16 g gut^−1^; five determinations), from the pyloric sphincter to the rectum including the modified fermentation sac, was placed into a 1.5-ml Eppendorf tube containing 0.5 ml of phosphate buffered saline. The hindgut sections of six individual larvae from each site or instar were pooled before DNA extraction.

### Extraction and purification of DNA

After mixing well, the hindgut samples were homogenized and centrifuged (13000×g for 5 min), and then the hindgut pellet was used for the DNA extraction. The DNA extractions were carried out using the enzymatic lysis protocol described in Yang *et al.* [33] with the following modifications: each pellet was suspended in 557 µl of Tris-EDTA (TE) buffer (100 mM Tris-HCl pH 7.5, 50 mM EDTA) and 10 µl lysozyme (238 mg mL^−1^) was added. After incubating at 37°C for 20 min, 3 µl proteinase K (20 mg mL^−1^) and 30 µl 10% sodium dodecyl sulfate were added. The mixture was then incubated for 1 h at 37°C. After the incubation step, 100 µl NaCl (5 mol L^−1^) and 80 µl CTAB/NaCl were added, followed by an incubation at 65°C for 10 min. The supernatant was then extracted using consecutive phenol-chloroform-isoamyl alcohol (25∶24∶1 by volume), isopropanol, and ethanol precipitation steps. Finally, the DNA was re-suspended in 100 µl of TE buffer and stored at −20°C until it was used for Polymerase Chain Reaction (PCR) and further analysis.

### PCR amplification of 16S rRNA gene

The variable V6-V8 region of the 16S rRNA gene was amplified using the DGGE primers F-968-GC and R-1401 [34]. The PCR was performed as previously described in Penders *et al.* [35], except that the annealing temperature was 58°C. Since PCR-based DNA techniques may introduce bias during PCR amplifications, it's very important to minimize PCR artifacts in clone library construction when study an environmental sample with high complex microbial diversity [36], [37]. In fact, we had performed a preliminary experiment to test whether a “reconditioning PCR” step can reduce the presence of heteroduplex sequences in our gut samples. After analyzing by DGGE, a weak band with novel DGGE mobility was observed in the products of common PCR protocol, But in products of PCR with reconditioning PCR step, no novel visible band was observed. So, “reconditioning PCR”, was performed as suggested by Acinas *et al.* [37] and Thompson *et al.* [38], to reduce PCR-produced biases and artifacts. After amplification, PCR products were checked by electrophoresis in 1.2% (w/v) agarose gels with subsequent ethidium bromide staining (10 mg mL^−1^).

### Construction of 16S rRNA gene libraries

Triplicate PCR products were pooled and cleaned using an *EasyPure* Quick Gel Extraction Kit (BeiJing TransGen Biotech Co., Ltd., BeiJing, China) following the manufacturer's instructions. Amplified gene fragments were cloned using the *p*EASY-T1 clone kit (BeiJing TransGen Biotech Co., Ltd., China) with blue-white selection. Approximately 90 white colonies were obtained from each of the three larval stages, and 170 white colonies were chosen from each natural population. Plasmid DNA from the cultured transformants was isolated by alkaline lysis [39], followed by PCR amplification using the M13 forward and reverse primers provided by the kit following manufacturer's instructions (*p*EASY-T1 clone kit, BeiJing TransGen Biotech Co., Ltd., China). For each clone library, positive clones that containing inserts of approximately 430 bp were selected and subsequently screened by DGGE.

### Screening of the 16S rRNA gene clone library by PCR and DGGE

The DGGE approach is widely used for monitoring bacterial growth and analyzing bacterial communities [28], [40], [41]. However, limitations of DGGE have also been pointed out by various authors, such as only bacterial populations that make up more than 1% of the total community can be detected [42], [43], [44], a complex community that comprised of numerous populations in relatively equivalent proportions will result in a smear of bands, which makes it difficult to identify individual populations and excise DGGE bands [45]. In order to overcome these deficiencies, we constructed the 16S rDNA clone libraries and screened the different clones by DGGE. This strategy combines the advantage of the original cloning method of the 16S rRNA gene with the advantage of DGGE, and allows screening different clones and analyzing the structure of the bacterial community in one gel.

**Figure 2:**
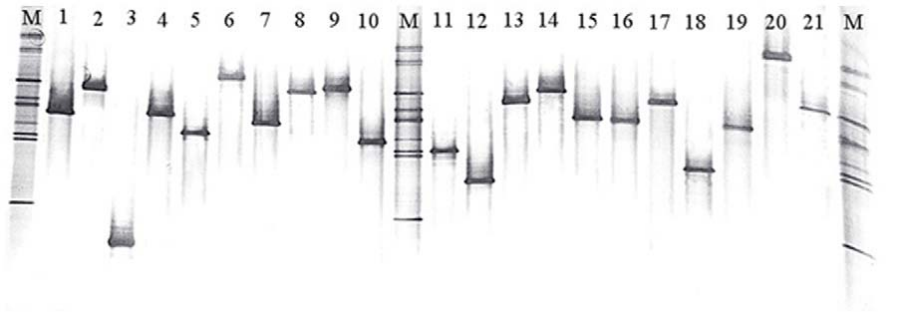
An image of representative gel showing DGGE analysis of PCR products amplified from individual clones. Lanes labeled 1 to 21 give examples of individual clones that were randomly picked out from the clone library of SC population. Clones with the same electrophoretic mobility in the gel were temporarily grouped into one group. The marker lanes (M) contained PCR-amplified 16S rRNA gene fragments of nine bacterial clones that showed different electrophoretic mobility in DGGE gel.

DGGE was performed using the DCode mutation detect system (Bio-Rad, USA). One-microliter PCR product of each positive clone that amplified by the M13 primer set in the above step was served as template DNA in a second PCR step using the F-968-GC and R1401 primers as described previously. Then 20 µl samples of the obtained PCR products were subsequently loaded on to 8% (w/v) polyacrylamide gels with a denaturing gradient of 30–70% (100% denaturant solution consists of 7 M urea and 40% formamide) and run for 20 h at 80 V at a constant temperature of 60°C in 0.5× Tris-Acetate-EDTA buffer (pH 7.4). To standardize the DGGE gels, a mixture of nine cloned DNA fragments with different electrophoretic positions was loaded as a marker. After electrophoresis, the DGGE bands were visualized with silver nitrate [46], and the DGGE gels were analyzed by GELCOMPARII software(Applied Maths NV, Belgium). Each clone library was analyzed individually, and clones with the same electrophoretic mobility in the gel were temporarily grouped into one group (Fig. 2). Once DGGE analysis was finished, the number of different groups in each clone library was recorded. The number of clones in each group was also recorded.

### Sequencing and Sequence analysis

For each clone library, a representative clone from each DGGE group was sequenced. All sequences were checked for chimeric artifacts using the check-chimera program of the Ribosomal Database Project (RDP) [47], and aligned against those found in the GenBank nr database, in the RDP II database [47], and on the EzTaxon server [48] using the Basic Local Alignment and Search Tool (BLAST) algorithm [49]. These nonchimeric sequences data have been submitted to the GenBank database under accession numbers JF964265–JF964858 and JN006162–JN006258.

All the sequences from the bacterial clone libraries were aligned with ClustalX(http://www.clustal.org/),and distance matrices were calculated according to the Jukes-Cantor distance Model in the PHYLIP 3.69 software package (http://evolution.gs.washington.edu/phylip.html) to group the sequences into operational taxonomic units (OTUs). Sequences were clustered by the program MOTHUR into OTUs using the furthest neighbor method and identity cut-offs of 0.03 representing approximately species-level clustering [50]. The OTU was also represented by the name of the representative clone in further phylogenetic analyses. In each clone library, if several groups were classified as the same OTU finally, the number of clones in these groups were added up to calculate the number of clones that this OTU contained. The output files from OTU clustering(distance matrices, file of representative OTU sequences) had been deposited to Data Dryad (http://datadryad.org) (doi:10.5061/dryad.h3r16).

### Phylogenetic analysis

Phylogenetic analysis of these partial 16S rRNA gene sequences was performed using the MEGA4.0 software [51]. For phylogenetic analysis, a representative clone of each OTU was chosen from the clone libraries, and their sequences were aligned with the nearest neighbors found in the NCBI database and SILVA web server [http://www.arbsilva. de/] [52]. Clones obtained from the larval gut of other scarab beetles that were available in NCBI database were also added to the alignment. Neighbour-joining (NJ) analysis was performed by using MEGA4.0 [51]. The NJ tree was constructed from the distance matrix calculated by the algorithm of Kimura's two-parameter model. Bootstrap confidence values were obtained with 1000 resamplings.

### Soil analyses and climatic data

Air-dried soil samples that had been sieved using a 2.0-mm sieve were used for analysis. Soil pH values were determined using a water (water: soil = 2.5∶1) suspension [53]. The total amount of organic carbon in the soil (hereafter “Total soil C”) was analyzed using the H_2_SO_4_-K_2_Cr_2_O_7_ oxidation method with an Alpkem autoanalyzer (Kjektec System 1026 Distilling. Unit, Sweden). The total amount of nitrogen in the soil (hereafter “Total soil N”) was measured using the Kjeldahl acid-digestion method. All of these analyses were completed in the Key Laboratory of Subtropical Agriculture and Environment, Ministry of Agriculture, in the Huazhong Agriculture University. The mean annual temperature (Annu), January low temperature (LowT), monthly temperature range (Ave, defined as the 12-month average of the differences between the monthly mean maximum and minimum temperatures), and mean annual precipitation (PRE) climatic data represent 40-year averages recorded between 1961 and 2000 at various locations throughout China. These data were obtained from http://cdc.cma.gov.cn, which is published by the China Meteorological Administration.

### Statistical analyses

The richness estimations and diversity indices were calculated using EstimateS (Version 8.2, http://viceroy.eeb.uconn.edu/EstimateS) software. The bias-corrected Chao1 estimator of species richness was calculated after 1,000 randomizations of sampling without replacement. The diversity of the sampled sequence set was estimated using the Simpson and Shannon indices (*H′*) in the EstimateS application. The coverage of the clone library is given as: C = 1−(n1/N), where n1 is the number of clones that occurred only once in the library, and N is the total number of clones examined [54]. Rarefaction curves were produced using the Analytic Rarefaction 1.3 program, which is available online at http://www.biology.ualberta.ca/jbrzusto/rarefact.php.

The correlation between the Chao1 estimates and environmental factors was analyzed using the Spearman nonparametric correlation test available in the SPSS 16.0 software (SPSS Inc., Chicago, IL).

∫-LIBSHUFF, a tool in the mothur software package that implements the Cramer-von Mises test statistic, was used to compare the clone libraries and determine the degree of similarity between them [50]. The relationship between the hindgut microbial community composition and environmental factors was analyzed by redundancy discriminate analysis (RDA) using CANOCO software (Microcomputer Power, Ithaca, New York). The data matrices containing the species data were log(x+1) transformed before analysis. Out of all of the environmental variables, the environmental factors that best described the most influential gradients were identified by manual forward selection and subjected to further analysis. We used a Monte Carlo permutation test based on 499 random permutations to test the significance (*P*<0.05) of the relationship between the explanatory variables and the community composition.

## Results

### Phylogenetic analyses of the dominant hindgut bacteria of *H. parallela*

A total of ten clone libraries were constructed to represent the bacterial communities naturally present in the hindgut of scarab beetle larvae. After DGGE analysis, 44–78 representative clones from each clone library (total 594 clones) were selected for sequencing (Table 1). None of the clones was discarded as chimeric, and 408 sequences (68.7% of the 594 sequenced clones) shared <97% sequence identity with published 16S rRNA gene sequences in the GenBank database. The 594 representative sequences were identified as *Firmicutes*, *Alpha-*, *Beta-*, *Gamma-* and *Deltaproteobacteria*, *Bacteroidetes*, *Actinobacteria* and *Fusobacteria* sequences. At a >97% sequence identity threshold, 205 OTUs could be defined. Most of the OTUs belong to *Firmicutes* (166 OTUs), *Deltaproteobacteria* (12 OTUs), *Gammaproteobacteria* (8 OTUs), and *Betaproteobacteria* (7 OTUs). The remaining groups were represented, to a much smaller extent, by five OTUs for *Bacteroidetes*, three OTUs for *Actinobacteria*, three OTUs for *Alphaproteobacteria*, and only one representative OTU for *Fusobacteria*.

**Figure 3:**
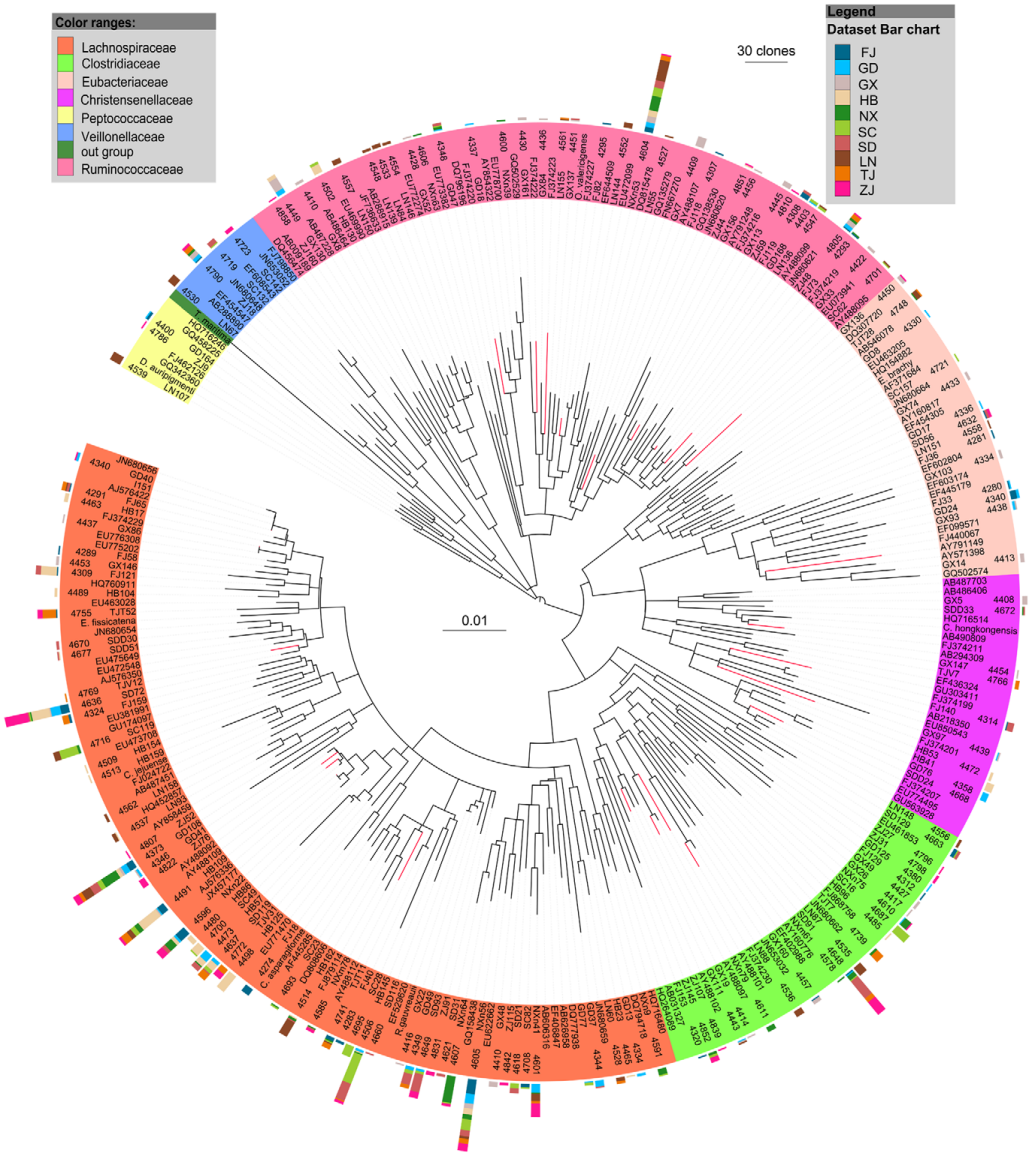
Neighbour-joining tree of all clones isolated from 10 *H. parallela* natural populations that affiliated to *Clostridia*. After construction, the tree was edited using the Interactive Tree of Life website (iTOL) [77]. The 16S rRNA gene sequence of *Thermotoga maritime* (AJ401021) was selected as an outgroup. Branches in red are clones obtained from other scarab larvae in previous studies. For clarity, clones were grouped into OTUs, with only 1 representative clone from each OTU included in the phylogenetic tree, and the last 4 numbers of their GenBANK accession numbers (JF964265–JF964858) were also included. Bar charts represent the relative number of clones obtained from each natural population. Bar in the center of the circle represents 0.01 substitutions per site. The abbreviations of the bacterial species were used are as follows: *R. gauvreauii*, *Ruminococcus gauvreauii* (EF529620); *C. jejuense*, *Clostridium jejuense* (AY494606); *E. brachy*, *Eubacterium brachy* (U13038); *D. auripigmenti*, *Desulfosporosinus auripigmenti* (AJ493051); *O. valericigenes*, *Oscillibacter valericigenes* (AP012044). *C. hongkongensis*, *Catabacter hongkongensis*(AB671763); *C. asparagiforme*, *Clostridium. asparagiforme* (ACCJ01000522); *E. fissicatena*, *Eubacterium fissicatena* (FR749937).

**Figure 4:**
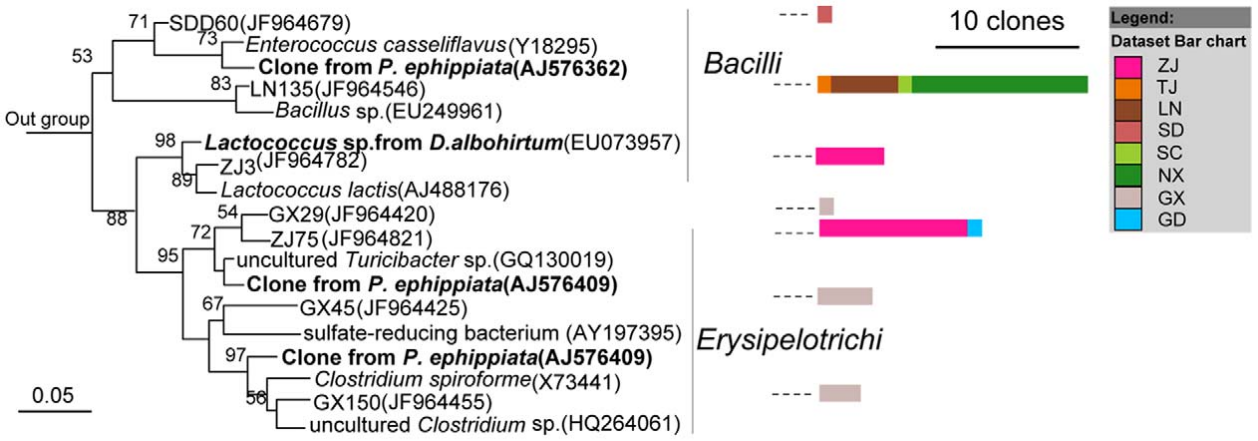
Neighbour-joining tree representing the Firmicutes-like bacterial clones isolated from *H. parallela* natural populations that besides *Clostridia*. Only 1 representative clone from each OTU was included in the phylogenetic tree. Bar charts represent the relative number of clones obtained from each natural population. The 16S rRNA gene sequence of *Cenarchaeum symbiosum* (U51469) was selected as an outgroup. Clones obtained in this study and closest relative bacterial clones that obtained previously from larvae of other scarab beetles, including *Pachnoda ephippiata* (*P. ephippiata*), *Dermolepida albohirtum* (*D. albohirtum*), and *Costelytra zealandica* (*C. zealandica*) were shown in bold. Bar charts represent the relative number of clones obtained from each natural population. Numbers at nodes are bootstrap support values ≥50%. The bar represents 0.05 substitutions per site respectively.

**Figure 5:**
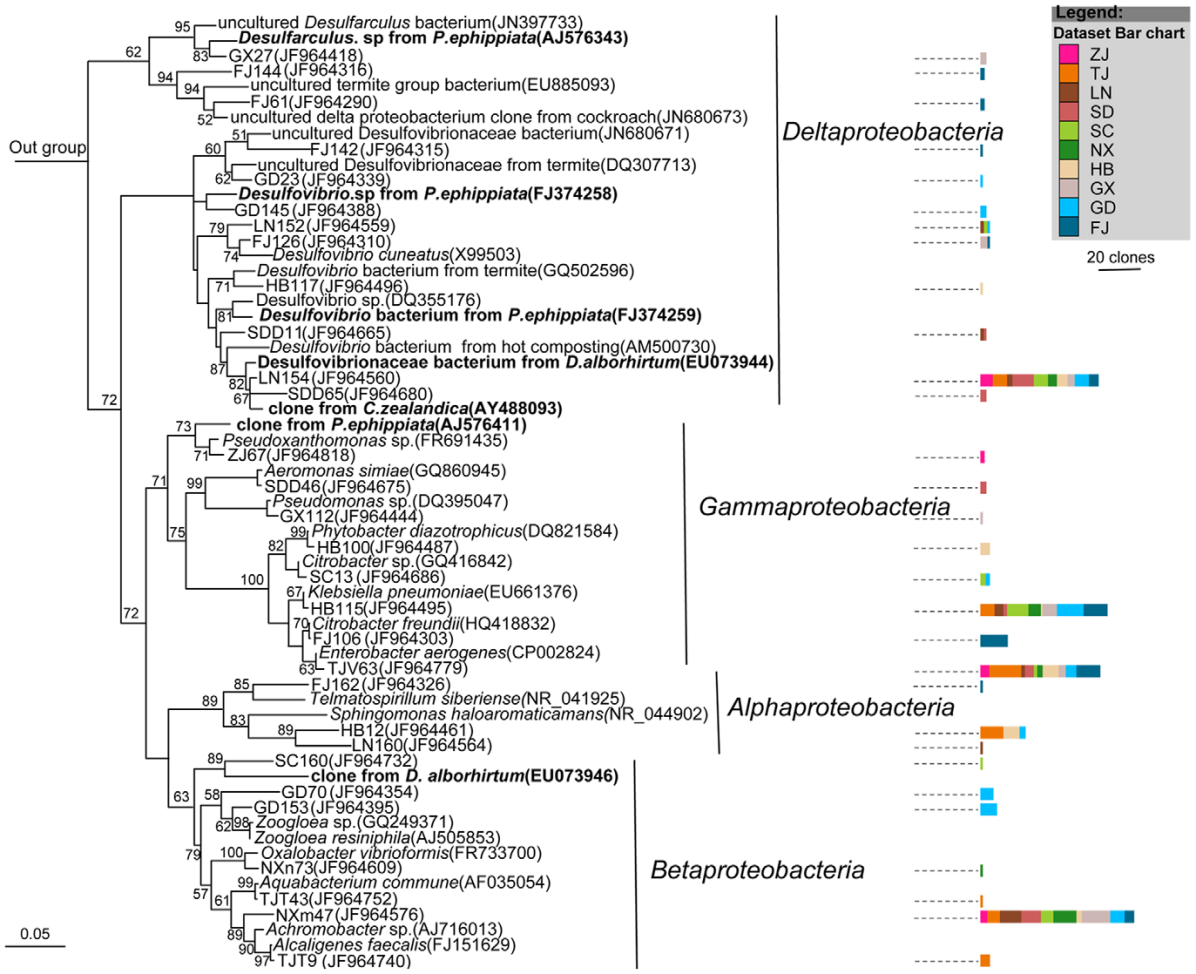
Neighbour-joining tree representing the *Proteobacteria*-like bacterial clones isolated from 10 *H. parallela* natural populations. Clones obtained in this study and closest relative bacterial clones that obtained previously form scarab beetle were shown in bold. Bar charts represent the relative number of clones obtained from each natural population. The bar represents 0.05 substitutions per site. For more information, see figure legends of Fig. 4.

**Figure 6:**
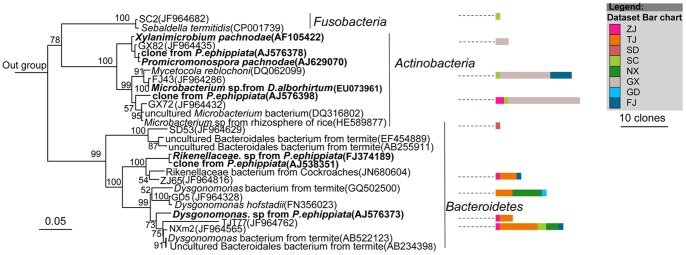
Neighbour-joining tree representing *Bacteroidetes*, *Fusobacteria*, and *Actinobacteria* bacterial clones isolated from *H. parallela* natural populations. Clones obtained in this study and closest relative bacterial clones that obtained previously form other scarab beetles were shown in bold. Bar charts represent the relative number of clones btained from each natural population. The bar represents 0.05 substitutions per site. For more information, see figure legends of Fig. 4.

Members of the largest group, *Firmicutes*, mostly belonged to the *Clostridia* class (Fig. 3), followed by *Bacilli* and *Erysipelotrichi* (Fig. 4). Most of the sequences belonging to *Clostridia* were affiliated with the *Lachnospiraceae*, *Ruminococcaceae*, *Clostridiaceae*, *Eubacteriaceae*, *Veillonellaceae*, and *Christensenellaceae* families (Fig. 3). The largest cluster was *Lachnospiraceae*, and clones in this cluster were closely related to several clones obtained previously from larvae of scarab beetle *Pachnoda ephippiata*, *Dermolepida albohirtum*, and *Costelytra zealandica*. Also, several clones were affiliated with *Clostridium jejuense* as the closest relative. Clones in another important cluster (*Ruminococcaceae*) was closely related to *Oscillibacter valericigenes*, and also comprised several clones obtained previously from the gut of the larvae of other scarab beetles (Fig. 3). The *Clostridiaceae* clones formed a distinct lineage among the *Clostridia* that contained no cultivated representatives. Their closest relatives were clones from other scarab beetles, termite guts and *Spodoptera littoralis*. Almost all of the OTUs of *Deltaproteobacteria* were grouped into the *Desulfovibrio* genus, and their closest relatives were *Desulfovibrio cuneatus* and some clones from scarab beetle guts (Fig. 5). The OTUs of *Gammaproteobacteria* were mostly related to the *Enterobacteriaceae* family, mainly the *Klebsiella*, *Enterobacter* and *Raoultella* genera (Fig. 5). All of the OTUs of the *Bacteroidetes* were related to the *Porphyromonadaceae* family. Several clones in this group were affiliated with *Dysgonomonas hofstadii* as the closest relative, while numerous other clones were closely related to clones from termite guts and other scarab beetles (Fig. 6). One *Actinobacteria* OTU that belonged to the *Promicromonosporaceae* family was identified as *Promicromonospora pachnodae* (99% sequence similarity), a dominant (hemi)cellulolytic bacterium found in the hindgut of the scarab beetle *Pachnoda marginata* (Cazemier *et al.*, 2003) (Fig. 6).

### Diversity analysis and structural comparison of gut microbiota

We evaluated the species richness and diversity of the bacterial communities in the hindgut of larvae of *H. parallela* from different sampling sites. Coverage values (ranging from 86.1% to 95.6%) and rarefaction curves (Fig. S1) indicated that most prevalent bacterial groups in the hindgut were identified. The number of OTUs identified from the different populations varied from 35 to 49 OTUs (Table 1). Species richness also varied across the host locations, as measured by the Chao1 estimator. The bacterial community from the TJ population (38.8) possessed the least species richness, while the FJ (86.5) population had the highest species richness. In addition, the Shannon diversity index (*H′*) in each clone library was high (ranging from 3.2 to 3.54), indicating a high diversity of bacterial phylotypes in the hindgut of *H. parallela*.

**Figure 7:**
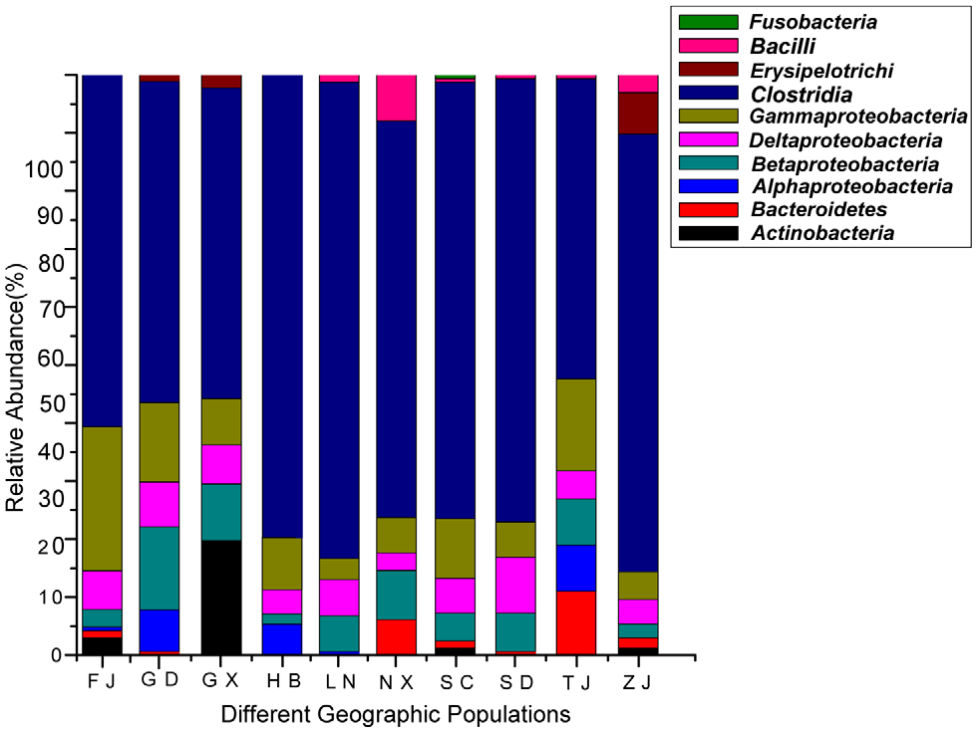
Relative abundance of different bacterial groups found in the hindgut of *H. parallela* from the 10 distinct natural populations.

One prominent feature of the gut microbiota of *H. parallela* larvae was the high prevalence of *Firmicutes* and *Proteobacteria* sequences (Fig. 7). Sequences affiliated with *Firmicutes* (mainly *Clostridia*) were detectable in all of the natural populations, and they were the most prominent group in the hindgut microbiota of the ten populations (ranging from 52% to 86.2%). Sequences related to *Beta-*, *Gamma-* and *Deltaproteobacteria* were also present in all natural populations, although at relatively low proportions (Fig. 7). However, even bacteria belonging to these major groups above, such as *Ruminococcaceae*, *Lachnospiraceae*, *Enterobacteriaceae*, and *Desulfovibrionaceae*, were commonly found in all of the natural populations, and constituted dominant and stable populations in the scarab gut, the composition and complexity of these dominant bacterial groups varied significantly among the ten populations. Only six OTUs (OTUNXn56, OTUNXn53, and OTUGD108 in the class *Clostridia*, OTUTJV63 in the class *Gammaproteobacteria*, OTUNXm47 in the class *Betaproteobacteria*, and OTULN154 in the class *Deltaproteobacteria*) were shared between the ten natural populations (Fig. 3 and Fig. 5); However, the remaining OTUs were unevenly spread across the host populations, with many appearing in only 1 host population among the ten sampled. In addition, bacteria belonging to other phyla (*Bacteroidetes*, *Actinobacteria*, and *Fusobacteria*) were also unevenly distributed across the host populations (Fig. 7). For example, *Bacteroidetes* were absent from the GX, HB, and LN populations, whereas bacteria belonging to the *Fusobacteria* were only detected in the SC population. The similarity indices calculated using the ∫-LIBSHUFF tool showed that the communities of the ten clone libraries are significantly different from each other (*P*<0.0011), with the exception of the clone library of the ZJ population, which showed no significant difference from the FJ and NX populations (Table S1).

### Influence of environmental factors on the hindgut bacterial community

The climate parameters and soil characteristics of the various locations sampled in this study are listed in Table 2. They revealed significant differences among the sampling regions. Positive correlations were found between the Chao1 estimate of richness and the Annu, PRE, and LowT measurements (Methods, Table 3). However, a negative correlation between the Chao1 estimate of richness and the soil pH (*r_s_* = −0.739, *p* = 0.015) was also observed. The Chao1 estimate of richness was not correlated with other environmental factors, including Ave, total soil N, total soil C and the C∶N ratio (Table 3).

**Table 2:** Climate data and soil characteristics obtained in this study.

site	Annu	Ave	PRE	LowT	Percent	Percent	C∶N	pH
	(°C)	(°C)	(mm)	(°C)	total N	carbon	ratio	
SC	16.3	7.4	921	2.7	0.12	0.93	7.75	7.6
NX	8.8	12.9	193	−13.5	0.14	1.39	9.29	5.9
GX	21.8	7.7	1320	10.1	0.20	2.06	10.3	6.7
GD	22.0	7.6	1682	10.1	0.09	1.06	11.78	6.7
HB	17.1	8.5	1242	−0.2	0.07	0.69	9.86	7.5
TJ	12.7	9.6	561	−7.4	0.12	0.99	8.25	8.5
FJ	21.4	5.8	1714	12.9	0.16	1.48	9.25	5.1
SD	12.4	6.2	720	−3.4	0.13	1.89	14.54	6.2
ZJ	16.0	6.0	1111	0.7	0.06	0.81	13.5	5.7
LN	7.9	11.9	684	−16.3	0.11	1.43	13	8.1

Abbreviations are used as follows: Annu, Mean annual temperature; Ave, monthly temperature range; PRE, mean annual precipitation; LowT, January low temperature; Total N, the total amount of nitrogen in the soil; Carbon, the total amount of organic carbon in the soil.

**Table 3:** Spearman correlations between Chao1 estimate of richness across host populations and the environmental factors.

Variable	*r_s_*	*p*
Annu	0.745	**0.013**
Ave	−0.515	0.128
PRE	0.718	**0.019**
LowT	0.855	**0.002**
Total N,%	0.17	0.638
Organic C, %	0.139	0.701
C∶N ratio	−0.125	0.731
pH	−0.739	**0.015**

Boldface values indicate significant *P* values (*P*<0.05). Abbreviations are used as follows: Annu, Mean annual temperature; Ave, monthly temperature range; PRE, mean annual precipitation; LowT, January low temperature; Total N, the total amount of nitrogen in the soil; Carbon, the total amount of organic carbon in the soil.

**Figure 8:**
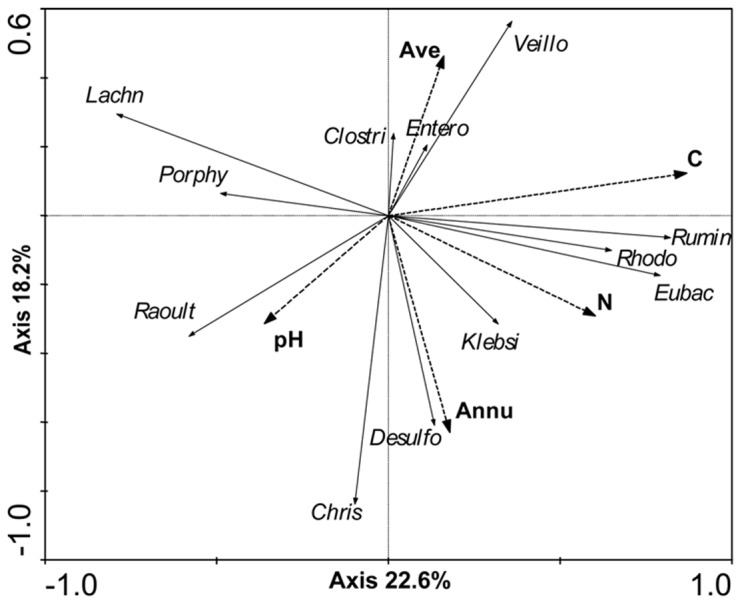
RDA biplots of selected (forward selection) environmental factors and the relative abundance of major bacterial groups in the hindgut of scarab larvae. The smaller the angle between the vectors (or a vector and an axis) and the longer the vectors, the more correlated are the variables represented by the vectors. The eigenvalues of the two axes are given in parentheses. Abbreviations used are as follows: Annu, Mean annual temperature; Ave, monthly temperature range; N, total nitrogen in the soil; C, total carbon in the soil; Rumin, *Ruminococcaceae*; Veillone, *Veillonellaceae*; Rhodo, *Rhodocyclaceae*, Clostri, *Clostridiaceae*; Klebsi, *Klebsiella*; Desulfo, *Desulfovibrio*; Eubac, *Eubacteriaceae*; Entero, *Enterobacter*; Raoult, *Raoultella*; Porphy, *Porphyromonadaceae*; Lachn, *Lachnospiraceae*, Chris, *Christensenellaceae*.

The RDA revealed that the bacterial community composition was related to environmental factors, including Ave, Annu, soil pH, total C and total N. The RDA yielded two main axes that explained 22.6% and 18.2%, respectively, of the total variation in bacterial community structure (Fig. 8). Biplot scaling of the RDA further indicated the influence of the different variables on the various bacterial phylotypes. The total C and total N of the soil correlated with the presence of the *Ruminococcaceae* family, *Eubacteriaceae* family and *Rhodocyclaceae* family. Correlations were also detected between the Ave and the *Clostridiaceae* family, *Veillonellaceae* family, and family *Enterobacteriaceae*. Additionally, the soil pH was correlated with the presence of the *Raoultella* genus, and Annu was correlated with the presence of the *Desulfovibrio* genus and *Klebsiella* genus. Additionally, there was a negative correlation of total N with the presence of bacteria from the *Lachnospiraceae* family, and the presence of the *Christensenellaceae* family were negatively correlated with Ave.

### Gut bacteria community in the larvae of different instars

Besides comparing the bacterial 16S rRNA gene sequence libraries recovered from ten natural populations, we also determined the gut microbial communities in the larvae of different instars. The purposes was to identifying the predominant bacterial species, possible trends in terms of microbial succession during larval development, and the presence of species that may have a symbiotic relationship with the scarab beetle.

**Figure 9:**
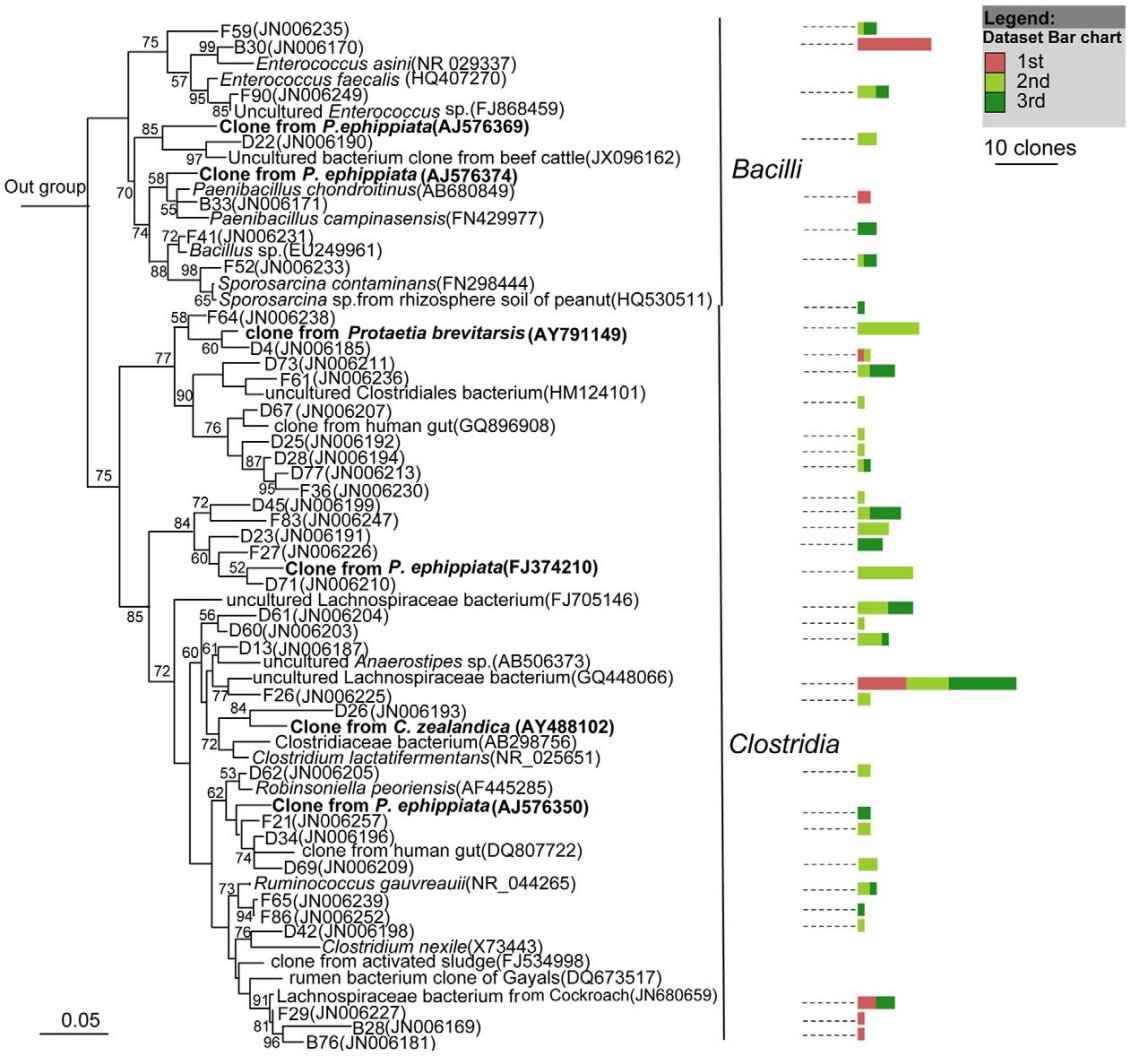
Neighbour-joining tree of *Firmicutes*-like bacterial clones isolated from different instars of *H. parallela*. Only 1 representative clone from each OTU was included in the phylogenetic tree. Clones obtained in this study and closest relative bacterial clones that obtained previously form other scarab beetles were shown in bold. Bar charts represent the relative number of clones obtained from each larval stage. The bar represents 0.05 substitutions per site. For more information, see figure legends of Fig. 4.

**Figure 10:**
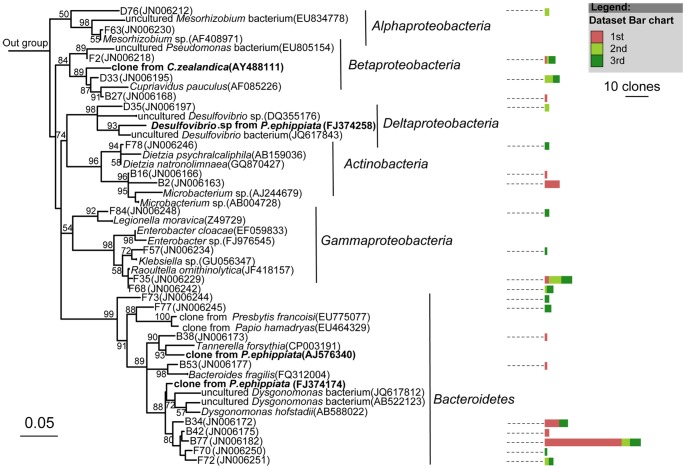
Neighbour-joining tree of *Bacteroidetes*, *Actinobacteria*, *Proteobacteria*bacterial clones isolated from different instars of *H. parallela*. Bar charts represent the relative number of clones obtained from each larval stage. Clones obtained in this study and closest relative bacterial clones that obtained previously form other scarab beetles were shown in bold. For other details, see figure legends of Fig. 4 and Fig. 9.

Overall, three 16S rRNA gene sequence libraries were constructed from all three instars. Approximately 30 representative clones from each clone library were sequenced and analyzed (Table 4). Fifty-eight OTUs over a broad diversity of bacteria were identified, including *Firmicutes*, *Proteobacteria*, *Actinobateria*, and *Bacteroidetes* (Fig. 9 and Fig. 10). Twenty-nine OTUs grouped with the *Clostridia*, and several clones in this group were affiliated with *Clostridium nexile*, *Ruminococcus gauvreauii*, and *Robinsoniella peoriensis* as the closest relative. Other *Clostridia* clones formed distinct lineages with no cultured representatives, and were closely related to the clones obtained from *P. ephippiata* and *Protaetia brevitarsis* (Fig. 9). OTUs affiliated with *Proteobacteria* were also dominant in the analyzed clones. Ten OTUs were grouped with the *Proteobacteria* (2 *Alphaproteobacteria*, 3 *Betaproteobacteria*, 4 *Gammaproteobacteria*, and 1 *Deltaproteobacteria* OTU) (Fig. 10). Nine OTUs were grouped within the *Bacteroidetes* (mainly *Porphyromonadaceae*), and seven OTUs within *Bacilli*. The remaining three OTUs were grouped within the *Actinobacteria* (Fig. 10).

**Table 4:** Characteristics of 16S rRNA gene sequence clone libraries and species richness estimates of different instars.

Instar	N^a^	N^b^	N^c^	Chao1	Simpson	Shannon(*H′*)	Coverage^d^
1st	88	23	17	27	7.67	2.04	88.6%
2nd	93	37	33	40.8	24.98	3.02	84.9%
3rd	88	36	31	39.3	25.72	2.89	83.0%

a Number of clones isolated from the host population.

b Number of representative clones that were chosen and sequenced.

c Number of OTUs identified in the 16S rRNA gene clone library. OTUs were defined based on 3% sequence divergence.

d library coverage calculated by the equation C = (1−n/N)*100, where n is the number of unique clones and N is the total number of clones examined.

**Figure 11:**
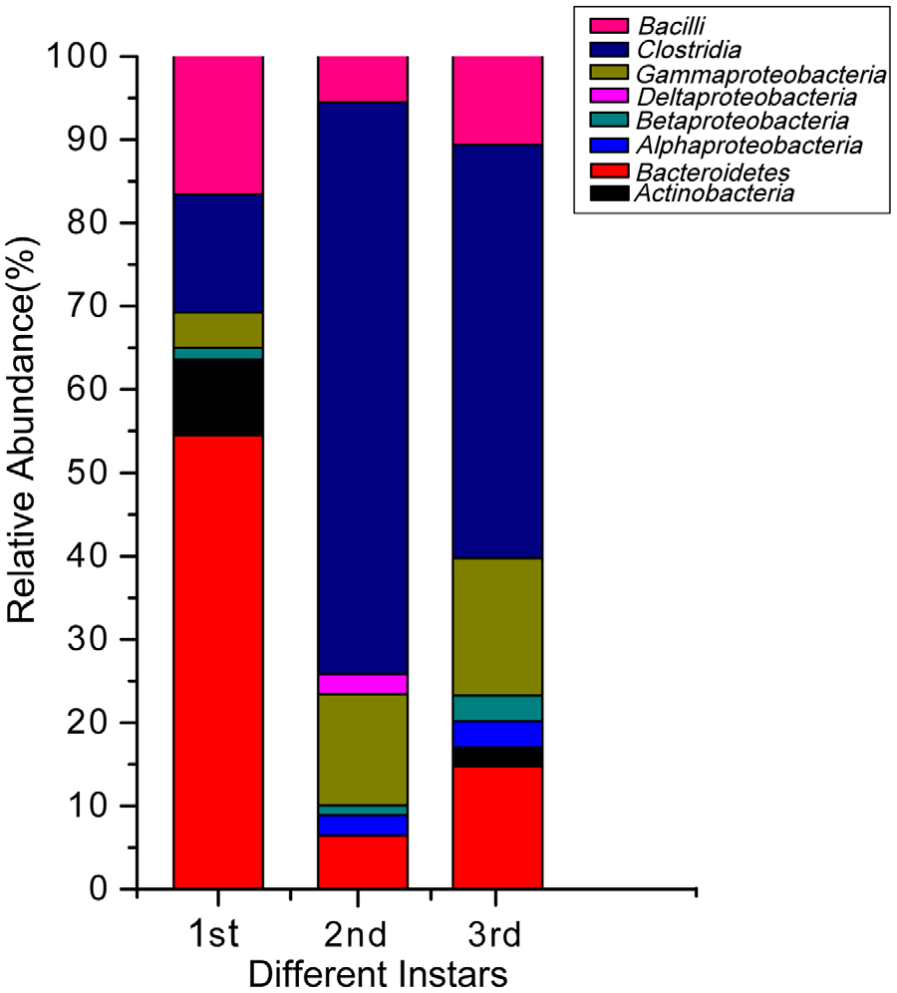
Relative abundance of different bacterial groups found in the hindgut of *H. parallela* at different instars.

Furthermore, significant changes in the complexity of the bacterial population during larval development were also observed (Fig. 9 and Fig. 10). The first instar larvae harbored a simpler bacterial community than the second and third instar larvae. *Bacteroidetes* were the mostly commonly found bacteria in the hindgut of the first instar larvae (48 clones, 54.55% of the first instar total clones) (Fig. 11), with one phylotype (represented by clone B77) predominantly found (37 clones, 42.05%). This OTU was also present with a low prevalence in the second- and third instar. Bacterial communities in second and third instar larvae were more complex, and they were dominated by the *Ruminococcaceae*, *Lachnospiraceae*, *Enterococcaceae* and *Enterobacteriaceae* families, whereas the *Bacteroidetes* phylum became a minor component of the community (Fig. 11). Overall, four phylotypes, including one *Porphyromonadaceae* (OTUB77), one *Pseudomonadaceae* (OTU F35), one *Rhodocyclaceae* (OTUF2), and one *Ruminococcaceae* (OTU F26), were present in the hindgut during all three instars, but at different frequencies. However, another 12 phylotypes were also shared by the second and third instar larvae, suggesting the compositions of the communities in the second and third instar larvae were similar to each other.

Although there are several OTUs in common between all three instars, the ∫-LIBSHUFF analysis revealed clear differences in the microbial community composition between the gut microbiota of first instar larvae and that from the second or third instar larvae (*P*<0.0083; Table S2). No significant differences were detected between the second and third instars. Chao1 estimator analysis (Table 4) and the rarefaction curves (Fig. S2) both showed that the hindgut bacteria communities in the second and third instar larvae were more diverse than those in the first instar larvae.

## Discussion

Using the DGGE technique and the analysis of the 16S rDNA clone libraries, we have identified bacteria that are associated with the *H. parallela* larval host over a broad geographic distribution. Diverse bacteria belonging to the *Firmicutes*, *Proteobacteria*, *Bacteroidetes*, *Actinobacteria* and *Fusobacteria* phyla were identified in the hindgut of *H. parallela* larvae, with the first two phyla dominating in all of the sampled populations. The microbial distribution of major phyla in the hindgut of *H. parallela* larvae was similar to that found in other scarabs, such as *Melolontha melolontha* [26], *D. albohirtum* [27], *C. zealandica* [28], and *P. ephippiata* [29]. Furthermore, many phylotypes from the hindgut of *H. parallela* larvae were closely related to the clones obtained from other scarab beetles (*P. ephippiata*; *D. albohirtum*, and *C. zealandica*), and some bacterial species even constituted monophyletic clusters (scarab beetle clusters) together with clones from other scarab beetles. These similarities suggest that although the bacterial community in the intestine of different scarab species is highly diverse, the phylogenetic placement of the taxa from the *Clostridia*, *Actinobacteria*, *Deltaproteobacteria* is correlated with each other. Sometimes these correlations are significant [27], and perhaps the presence of the taxon-specific lineages is indicative of cospeciation between gut microbiota and the host [29].

One prominent feature of the gut microbiota of *H. parallela* larvae was the high prevalence of *Firmicutes* sequences, which is similar to previous studies on the gut microbiota of other scarab beetles *M. melolontha* [26], *C. zealandica* [28], and *P. ephippiata* [29]. *Firmicutes* have been broadly found in the intestine of iguanas and mammals, and these bacteria are thought to have important and core roles in the host's metabolism [55], [56]. Microbial symbionts, especially the clostridial bacteria, are responsible for the metabolism of cellulose in the bamboo diet of pandas [57]. This situation also appears to apply to the *Scarabaeidae* family. *Firmicutes* contain many cellulolytic *Bacillus* and *Clostridium* species. The cellulose and hemicelluloses in roots that are ingested by the scarab larvae can first be hydrolyzed by *Firmicutes* to their constituent hexoses and pentoses, which can be further fermented to volatile fatty acids (VFA) or other by-products usable by methanogenic bacteria [30]. Additionally, *Clostridia-* and *Bacteroides*-related bacteria are responsible for the formation of butyrate, a characteristic product in the hindgut contents of *P. ephippiata* [31]. Functions have been suggested for some of the other bacterial groups detected in this study. Members of the *Enterobacteriaceae* family have involved in nitrogen and carbon metabolism in fruit fly Ceratitis capitata, and had an indirect contribution to host fitness [10]. A recent study showed that *Desulfovibrio* spp. were the most prominent group of microorganisms located almost exclusively in the gut periphery of *M. melolontha*, and played an important physiological role of sulfate metabolism of the host [26]. Interestingly, in this study, six bacterial species were consistently detected in all of the natural populations, suggesting the existence of a symbiotic relationship between these bacteria and the *H. parallela* larvae. This evidence further suggests that hindgut bacteria may fulfill important primary functions in the scarab beetle.

The diversity and composition of the hindgut bacterial communities varied significantly between larvae from different geographic location. The bacterial species richness significantly correlated with LowT, Annu, PRE and soil pH. Additionally, the composition and complexity of those dominant bacterial groups were also significantly affected by Ave, Annu and soil characteristics (i.e., pH, total C, total N), suggesting that the environment inhabited by the scarabs can influence the diversity of their gut bacterial communities. Our results are similar to those found in previous studies [17], [58], [59], [60]. Tsuchida *et al.* [58] reported a significant positive correlation between the pea aphid U-type symbiont (PAUS) infection and the mean annual temperature and mean annual precipitation. Previous research showed that the prevalence of symbionts in the stinkbugs *Acrosternum hilare* and *Murgantia histrionica* was significantly influenced by temperature [60]. Zouache *et al.* [17] found that habitats can affect the bacterial diversity of wild *Aedes* populations. Because insects are small-bodied ectotherms, their life cycle duration, survival, and behavior are dependent on ambient temperature [61], [62]. A warm climate might favor the survival and reproduction of bacteria in the insect's gut. Therefore, it is appropriate that bacterial species richness was significantly correlated with LowT and Annu.

In addition, scarab larvae live in the soil where they feed on organic matter. The soil characteristics and the soil microbial community may also affect the bacterial composition of scarab larvae's gut, and they may have caused the spatial changes in the bacterial communities that were seen in this study. Previous studies on the gut microbes of sand fly *Phlebotomus argentipes* [63], zebrafish [64] and the symbionts of the stinkbug *Riptortus clavatus* [65] have shown a strong correlation between insect-microbial associations and the environment in which they reside. Furthermore, it has been shown that soil chemistry characteristics [66], [67], soil moisture, and soil temperature [68], [69] markedly affect soil bacterial composition and diversity. Previous studies have also shown that plant species and agricultural management regime can influence the activity and composition of soil bacterial communities [22], [41], [70]. Although most of the sampling sites were peanut fields, the management regime of these fields were different. For example, wheat-corn rotation was the main agricultural management regime where the NX population was collected, but the field that the GX population was collected has been exposed to spring soybean-peanut rotation for several years. Moreover, the GD population and ZJ population were collected from fields subjected to rice-peanut rotation. The difference of agricultural practices in these sampling sites can also have impacts on the bacterial community structure in the soil and were responsible for the bacterial community variations we recorded. However, in this study, we found that some environmental factors that can affect the soil bacterial community, such as soil pH, total C and mean annual precipitation (which can affect soil moisture), also affected the constitution and diversity of the bacterial community of the scarab larvae's gut. This result not only indicates that some of the bacterial species that colonize the gut of scarab beetles might originate from the surrounding soil, but it also highlights, as suggested by Zouache *et al.* [17], the importance of considering environmental factors (i.e., ecological niches and ecoclimatic characteristics) when analyzing the symbiotic microbiota associated with wild animal populations.

Our results also suggest that the host developmental stage affects the composition of the hindgut bacterial community. The first instar larvae of *H. parallela* have microbiota with lower diversity, and the hindgut microbiota is dominated by *Bacteroidales*. However, in the second- and third instar, there were more diverse bacteria species, and the hindgut microbiota during these instars are dominated by several phylotypes such as *Clostridiales* and *Lactobacillales*. Such shifts in the dominant gut bacterial species between different life stages have also been detected in *Macrotermes gilvus* [71], *Anopheles gambiae* [72], *Frankliniella occidentalis* [73], and *Wohlfahrtia magnifica* [74]. The amount of feeding is usually low during the first instar, and it increases during larval development or body size increase [75], [76]. The variation in the gut bacterial community might be due, to some extent, to the food source and the digestion process, as suggested by Hongoh *et al.* [71]. The second and third instar larvae require greater amounts of food than first instar larvae. The increased number of fermentative bacteria groups, like *Lactobacillales* and *Clostridiales*, during the second and third instar larvae indicates that these bacteria might be involved in the fermentation process of the food in the gut of the larvae. Alternatively, the animal gut pH, redox conditions, and digestive enzymes present also have significant effects on the gut microbial community [1]. Therefore, it is possible that the hindgut bacterial community shift correlates with changes of gut physiology during larval development.

## Supporting Information

Figure S1 **Rarefaction analysis of bacterial 16S rRNA gene clone libraries recovered from 10** ***H. parallela*** **natural populations.** The predicted number of OTUs was calculated at the 3% level of sequence divergence.
(TIF)

Figure S2 **Rarefaction analysis of bacterial 16S rRNA gene clone libraries: recovered from different larval stages.** The predicted number of OTUs was calculated at the 3% level of sequence divergence.
(TIF)

Table S1 (DOC)

Table S2 (DOC)

## References

[1] Dillon RJ, Dillon VM (2004) The gut bacteria of insects: nonpathogenic interactions. Annu Rev Entomol 49: 71–92. 14651457 10.1146/annurev.ento.49.061802.123416

[2] Moran NA, McCutcheon JP, Nakabachi A (2008) Genomics and Evolution of Heritable Bacterial Symbionts. Annu Rev Genet 42: 165–190. 18983256 10.1146/annurev.genet.41.110306.130119

[3] Moya A, Peretó J, Gil R, Latorre A (2008) Learning how to live together: genomic insights into prokaryote–animal symbioses. Nat Rev Genet 9: 218–229. 18268509 10.1038/nrg2319

[4] Watanabe H, Tokuda G (2010) Cellulolytic systems in insects. Annu Rev Entomol 55: 609–632. 19754245 10.1146/annurev-ento-112408-085319

[5] Breznak JA, Brune A (1994) Role of Microorganisms in the Digestion of Lignocellulose by Termites. Annu Rev Entomol 39: 453–487.

[6] Douglas AE (1998) Nutritional interactions in insect-microbial symbioses: aphids and their symbiotic bacteria *Buchnera* . Annu Rev Entomol 43: 17–37. 15012383 10.1146/annurev.ento.43.1.17

[7] Hosokawa T, Kikuchi Y, Nikoh N, Shimada M, Fukatsu T (2006) Strict host-symbiont cospeciation and reductive genome evolution in insect gut bacteria. PLoS Biol 4 (10) e337 DOI: 10.1371/journal.pbio.0040337. 17032065 10.1371/journal.pbio.0040337 PMC1592312

[8] Visôtto LE, Oliveira MG, Guedes RN, Ribon AO, Good-God PI (2009) Contribution of gut bacteria to digestion and development of the *velvetbean caterpillar*, *Anticarsia gemmatalis* . J Insect Physiol 55: 185–191. 19061893 10.1016/j.jinsphys.2008.10.017

[9] DeVries EJ, Jacobs G, Sabelis MW, Menken SBJ, Breeuwer JAJ (2004) Diet-dependent effects of gut bacteria on their insect host: the symbiosis of *Erwinia* sp. and western flower thrips. Proc R Soc B: Biol Sci 271: 2171–2178. 10.1098/rspb.2004.2817 PMC1691834 15475338

[10] Behar A, Yuval B, Jurkevitch E (2008) Gut bacterial communities in the Mediterranean fruit fly (*Ceratitis capitata*) and their impact on host longevity. J Insect Physiol 54: 1377–1383. 18706909 10.1016/j.jinsphys.2008.07.011

[11] Oliver KM, Russell JA, Moran NA, Hunter MS (2003) Facultative bacterial symbionts in aphids confer resistance to parasitic wasps. Proc Natl Acad Sci U S A 100: 1803–1807. 12563031 10.1073/pnas.0335320100 PMC149914

[12] Dillon RJ, Vennard CT, Buckling A, Charnley AK (2005) Diversity of locust gut bacteria protects against pathogen invasion. Ecol Lett 8: 1291–1298.

[13] Dowd PF (1992) Insect fungal symbionts: A promising source of detoxifying enzymes. J Indust Microbiol Biotechnol 9: 149–161.

[14] Piel J (2002) A polyketide synthase-peptide synthetase gene cluster from an uncultured bacterial symbiont of Paederus beetles. Proc Natl Acad Sci U S A 99: 14002–14007. 12381784 10.1073/pnas.222481399 PMC137826

[15] Dillon RJ, Vennard CT, Charnley AK (2002) A Note: Gut bacteria produce components of a locust cohesion pheromone. J Appl Microbiol 92: 759–763. 11966918 10.1046/j.1365-2672.2002.01581.x

[16] Koch H, Schmid-Hempel P (2011) Bacterial communities in central European bumblebees: low diversity and high specificity. Microb Ecol 62: 121–133. 21556885 10.1007/s00248-011-9854-3

[17] Zouache K, Raharimalala FN, Raquin V, Tran-Van V, Raveloson LH, et al (2011) Bacterial diversity of field-caught mosquitoes, *Aedes albopictus* and *Aedes aegypti*, from different geographic regions of Madagascar. FEMS Microbiol Ecol 75: 377–389. 21175696 10.1111/j.1574-6941.2010.01012.x

[18] Jiao N, Zhang Y, Zeng Y, Hong N, Liu R, et al (2007) Distinct distribution pattern of abundance and diversity of aerobic anoxygenic phototrophic bacteria in the global ocean. Environ Microbiol 9: 3091–3099. 17991036 10.1111/j.1462-2920.2007.01419.x

[19] Ghiglione JF, Palacios C, Marty JC, Mével G, Labrune C, et al (2008) Role of environmental factors for the vertical distribution (0–1000 m) of marine bacterial communities in the NW Mediterranean Sea. Biogeosciences 5: 1751–1764.

[20] Sheik CS, Beasley WH, Elshahed MS, Zhou X, Luo Y, et al (2011) Effect of warming and drought on grassland microbial communities. ISME J 5: 1692–1700. 21451582 10.1038/ismej.2011.32 PMC3176507

[21] da C Jesus E, Marsh TL, Tiedje JM, de S Moreira FM (2009) Changes in land use alter the structure of bacterial communities in Western Amazon soils. ISME J 3: 1004–1011. 19440233 10.1038/ismej.2009.47

[22] Knief C, Ramette A, Frances L, Alonso-Blanco C, Vorholt JA (2010) Site and plant species are important determinants of the Methylobacterium community composition in the plant phyllosphere. ISME J 4: 719–728. 20164863 10.1038/ismej.2010.9

[23] Lavelle P, Bignell D, Lepage M, Wolters V, Roger P, et al (1997) Soil function in a changing world: the role of invertebrate ecosystem engineers. Eur J Soil Biol 33: 159–193.

[24] Cazemier AE, Verdoes JC, Reubsaet FA, Hackstein JH, Drift Cv, et al (2003) *Promicromonospora pachnodae* sp. nov., a member of the (hemi)cellulolytic hindgut flora of larvae of the scarab beetle *Pachnoda marginata* . Antonie Van Leeuwenhoek 83: 135–148. 12785307 10.1023/a:1023325817663

[25] Egert M, Wagner B, Lemke T, Brune A, Friedrich MW (2003) Microbial community structure in midgut and hindgut of the humus-feeding larva of *Pachnoda ephippiata* (Coleoptera: Scarabaeidae). Appl Environ Microbiol 69: 6659–6668. 14602626 10.1128/AEM.69.11.6659-6668.2003 PMC262301

[26] Egert M, Stingl U, Bruun DL, Wagner B, Brune A, et al (2005) Structure and topology of microbial communities in the major gut compartments of *Melolontha melolontha* larvae (Coleoptera: Scarabaeidae). Appl Environ Microbiol 71: 4556–4566. 16085849 10.1128/AEM.71.8.4556-4566.2005 PMC1183286

[27] Pittman GW, Brumbley SM, Allsopp PG, O'Neill SL (2008) “*Endomicrobia*” and other bacteria associated with the hindgut of *Dermolepida albohirtum* larvae. Appl Environ Microbiol 74: 762–767. 18083861 10.1128/AEM.01831-07 PMC2227725

[28] Zhang HY, Jackson TA (2008) Autochthonous bacterial flora indicated by PCR-DGGE of 16S rRNA gene fragments from the alimentary tract of *Costelytra zealandica* (Coleoptera: Scarabaeidae). J Appl Microbiol 105: 1277–1285. 18713286 10.1111/j.1365-2672.2008.03867.x

[29] Andert J, Marten A, Brandl R, Brune A (2010) Inter- and intraspecific comparison of the bacterial assemblages in the hindgut of humivorous scarab beetle larvae (*Pachnoda* spp.). FEMS Microbiol Ecol 74: 439–449. 20738398 10.1111/j.1574-6941.2010.00950.x

[30] Bayon C (1980) Volatile fatty acids and methane production in relation to anaerobic carbohydrate fermentation in *Oryctes nasicornis* larvae (Coleoptera: Scarabaeidae). J Insect Physiol 26: 819–828.

[31] Lemke T, Stingl U, Egert M, Friedrich MW, Brune A (2003) Physicochemical conditions and microbial activities in the highly alkaline gut of the humus-feeding larva of *Pachnoda ephippiata* (Coleoptera: Scarabaeidae). Appl Environ Microbiol 69: 6650–6658. 14602625 10.1128/AEM.69.11.6650-6658.2003 PMC262302

[32] Zhou LM, Ju Q, Qu MJ, Zhao ZQ, Dong SL, et al (2009) EAG and behavioral responses of the large black chafer, *Holotrichia parallela* (Coleoptera: Scarabaeidae) to its sex pheromone. Acta Entomol Sin 52: 121–125.

[33] Yang H, Schmitt-Wagner Dirk, Stingl U, Brune A (2005) Niche heterogeneity determines bacterial community structure in the termite gut (*Reticulitermes santonensis*). Environ Microbiol 7: 916–932. 15946289 10.1111/j.1462-2920.2005.00760.x

[34] Nübel U, Engelen B, Felske A, Snaidr J, Wieshuber A, et al (1996) Sequence heterogeneities of genes encoding 16S rRNAs in *Paenibacillus polymyxa* detected by temperature gradient gel electrophoresis. J Bacteriol 178: 5636–5643. 8824607 10.1128/jb.178.19.5636-5643.1996 PMC178401

[35] Penders J, Stobberingh EE, Thijs C, Adams H, Vink C, et al (2006) Molecular fingerprinting of the intestinal microbiota of infants in whom atopic eczema was or was not developing. Clin Exp Allergy 36: 1602–1608. 17177684 10.1111/j.1365-2222.2006.02599.x

[36] Suzuki MT, Giovannoni SJ (1996) Bias caused by template annealing in the amplification of mixtures of 16S rRNA genes by PCR. Appl Environ Microbiol 62: 625–630. 8593063 10.1128/aem.62.2.625-630.1996 PMC167828

[37] Acinas SG, Sarma-Rupavtarm R, Klepac-Ceraj V, Polz MF (2005) PCR-induced sequence artifacts and bias: insights from comparison of two 16S rRNA clone libraries constructed from the same sample? Appl Environ Microbiol 72: 8966–8969. 10.1128/AEM.71.12.8966-8969.2005 PMC1317340 16332901

[38] Thompson JR, Marcelino LA, Polz MF (2002) Heteroduplexes in mixed-template amplifications: formation, consequence and elimination by ‘reconditioning PCR’. Nucleic Acids Res 30: 2083–2088. 11972349 10.1093/nar/30.9.2083 PMC113844

[39] Sambrook J, Fritsch EF, Maniatis T (1989) Molecular cloning: A laboratory manual, 2nd edition. New York : Cold Spring Harbor Laboratory Press, Cold Spring Harbor.

[40] Liu WT, Huang CL, Hu JY, Song L, Ong SL, et al (2002) Denaturing Gradient Gel Electrophoresis Polymorphism for Rapid 16s rDNA Clone Screening and Microbial Diversity Study. J Biosci Bioeng 93: 101–103.

[41] Patra AK, Abbadie L, Clays-Josserand A, Degrange V, Grayston SJ, et al (2006) Effects of management regime and plant species on the enzyme activity and genetic structure of N-fixing, denitrifying and nitrifying bacterial communities in grassland soils. Environ Microbiol 8: 1005–1016. 16689721 10.1111/j.1462-2920.2006.00992.x

[42] Muyzer G, deWaal EC, Uitterlinden AG (1993) Profiling of complex microbial populations by denaturing gradient gel electrophoresis analysis of polymerase chain reaction-amplified genes coding for 16S rRNA. Appl Environ Microbiol 59: 695–700. 7683183 10.1128/aem.59.3.695-700.1993 PMC202176

[43] Murray AE, Hollibaugh JT, Orrego C (1996) Phylogenetic compositions of bacterioplankton from two California estuaries compared by denaturing gradient gel electrophoresis of 16S rDNA fragments. Appl Environ Microbiol 62: 2676–2680. 8779608 10.1128/aem.62.7.2676-2680.1996 PMC168051

[44] Kirk JL, Klironomos JN, Lee H, Trevors JT (2005) The effects of perennial ryegrass and alfalfa on microbial abundance and diversity in petroleum contaminated soil. Environ Pollut 133: 455–465. 15519721 10.1016/j.envpol.2004.06.002

[45] Nakatsu CH, Torsvik V, Øvreås L (2000) Soil Community Analysis Using DGGE of 16S rDNA Polymerase Chain Reaction Products. Soil Sci Soc Am J 64: 1382–1388.

[46] Sanguinetti CJ, Neto ED, Simpson AJ (1994) Rapid silver staining and recovery of PCR products separated on polyacrylamide gels. Biotechniques 17: 914–921. 7840973

[47] Cole JR, Wang Q, Cardenas E, Fish J, Chai B, et al (2009) The Ribosomal Database Project: improved alignments and new tools for rRNA analysis. Nucleic Acids Res 37: D141–D145. 19004872 10.1093/nar/gkn879 PMC2686447

[48] Chun J, Lee JH, Jung Y, Kim M, Kim S, et al (2007) Eztaxon: A web-based tool for the identification of prokaryotes based on 16s ribosomal rna gene sequences. Int J Syst Evol Microbiol 57: 2259–2261. 17911292 10.1099/ijs.0.64915-0

[49] Altschul SF, Gish W, Miller W, Myers EW, Lipman DJ (1990) Basic local alignment search tool. J Mol Biol 215: 403–410. 2231712 10.1016/S0022-2836(05)80360-2

[50] Schloss PD, Westcott SL, Ryabin T, Hall JR, Hartmann M, et al (2009) Introducing mothur: Open Source, Platform-independent, Community-supported Software for Describing and Comparing Microbial Communities. Appl Environ Microbiol 75: 7537–7541. 19801464 10.1128/AEM.01541-09 PMC2786419

[51] Kumar S, Dudley J, Nei M, Tamura K (2008) MEGA: A biologist-centric software for evolutionary analysis of DNA and protein sequences. Brief Bioinform 9: 299–306. 18417537 10.1093/bib/bbn017 PMC2562624

[52] Pruesse E, Quast C, Knittel K, Fuchs BM, Ludwig W, et al (2007) SILVA: a comprehensive online resource for quality checked and aligned ribosomal RNA sequence data compatible with ARB. Nucleic Acids Res 35: 7188–7196. 17947321 10.1093/nar/gkm864 PMC2175337

[53] Cayley GR, Lord KA (1980) The extraction and assay of thiabendazole in strongly adsorbing soils. Pestic Sci 11: 9–14.54.

[54] Good IJ (1953) The Population Frequencies of Species and the Estimation of Population Parameters. Biometrika 40: 237–264.

[55] Ley RE, Lozupone CA, Hamady M, Knight R, Gordon JI (2008) Worlds within worlds: evolution of the vertebrate gut microbiota. Nat Rev Microbiol 6: 776–788. 18794915 10.1038/nrmicro1978 PMC2664199

[56] Hong P-Y, Wheeler E, Cann IKO, Mackie RI (2011) Phylogenetic analysis of the fecal microbial community in herbivorous land and marine iguanas of the Galápagos Islands using 16S rRNA-based pyrosequencing. ISME J 5: 1461–1470. 21451584 10.1038/ismej.2011.33 PMC3160690

[57] Zhu L, Wu Q, Dai J, Zhang S, Wei F (2011) Evidence of cellulose metabolism by the giant panda gut microbiome. Proc Natl Acad Sci U S A 108: 17714–17719. 22006317 10.1073/pnas.1017956108 PMC3203778

[58] Tsuchida T, Koga R, Shibao H, Matsumoto T, Fukatsu T (2002) Diversity and geographic distribution of secondary endosymbiotic bacteria in natural populations of the pea aphid, *Acyrthosiphon pisum* . Mol Ecol 11: 2123–2135. 12296954 10.1046/j.1365-294x.2002.01606.x

[59] Behar A, Yuval B, Jurkevitch E (2008) Community structure of the Mediterranean fruit fly microbiota: Seasonal and spatial sources of variation. Isr J Ecol Evol 53: 181–191.

[60] Prado SS, Hung KY, Daugherty MP, Almeida RPP (2010) Indirect Effects of Temperature on Stink Bug Fitness, via Maintenance of Gut-Associated Symbionts. Appl Environ Microbiol 76: 1261–1266. 20023083 10.1128/AEM.02034-09 PMC2820946

[61] Tun-Lin W, Burkot TR, Kay BH (2000) Effects of temperature and larval diet on development rates and survival of the dengue vector *Aedes aegypti* in north Queensland, Australia. Med Vet Entomol 14: 31–37. 10759309 10.1046/j.1365-2915.2000.00207.x

[62] Delatte H, Gimonneau G, Triboire A, Fontenille D (2009) Influence of temperature on immature development, survival, longevity, fecundity, and gonotrophic cycles of *Aedes albopictus*, vector of chikungunya and dengue in the Indian Ocean. J Med Entomol 46: 33–41. 19198515 10.1603/033.046.0105

[63] Hillesland H, Read A, Subhadra B, Hurwitz I, McKelvey R, et al (2008) Identification of Aerobic Gut Bacteria from the Kala Azar Vector, *Phlebotomus argentipes*: A Platform for Potential Paratransgenic Manipulation of Sand Flies. Am J Trop Med Hyg 79: 881–886. 19052297

[64] Roeselers G, Mittge EK, Stephens WZ, Parichy DM, Cavanaugh CM, et al (2011) Evidence for a core gut microbiota in the zebrafish. ISME J 5: 1595–1608. 21472014 10.1038/ismej.2011.38 PMC3176511

[65] Kikuchi Y, Hosokawa T, Fukatsu T (2007) Insect-microbe mutualism without vertical transmission: a stinkbug acquires a beneficial gut symbiont from the environment every generation. Appl Environ Microbiol 73: 4308–4316. 17483286 10.1128/AEM.00067-07 PMC1932760

[66] Hartmana WH, Richardsona CJ, Vilgalysb R, Brulandc GL (2008) Environmental and anthropogenic controls over bacterial communities in wetland soils. Proc Natl Acad Sci U S A 105: 17842–17847. 19004771 10.1073/pnas.0808254105 PMC2584698

[67] deGraaff MA, Classen AT, FCastro H, Schadt CW (2010) Labile soil carbon inputs mediate the soil microbial community composition and plant residue decomposition rates. New Phytol 188: 1055–1064. 21058948 10.1111/j.1469-8137.2010.03427.x

[68] Bell C, McIntyre N, Cox S, Tissue D, Zak J (2008) Soil Microbial Responses to Temporal Variations of Moisture and Temperature in a Chihuahuan Desert Grassland. Microb Ecol 56: 153–167. 18246293 10.1007/s00248-007-9333-z

[69] Clark JS, Campbell JH, Grizzle H, Acosta-Martìnez V, Zak JC (2009) Soil microbial community response to drought and precipitation variability in the Chihuahuan Desert. Microb Ecol 57: 248–260. 19067031 10.1007/s00248-008-9475-7

[70] Salles JF, van Elsas JD, van Veen JA (2006) Effect of agricultural management regime on *Burkholderia* community structure in soil. Microb Ecol 52: 267–279. 16897309 10.1007/s00248-006-9048-6

[71] Hongoh Y, Ekpornprasit L, Inoue T, Moriya S, Trakulnaleamsai S, et al (2006) Intracolony variation of bacterial gut microbiota among castes and ages in the fungus-growing termite *Macrotermes gilvus* . Mol Ecol 15: 505–516. 16448416 10.1111/j.1365-294X.2005.02795.x

[72] Briones AM, Shililu J, Githure J, Novak R, Raskin L (2008) *Thorsellia anophelis* is the dominant bacterium in a Kenyan population of adult *Anopheles gambiae* mosquitoes. ISME J 2: 74–82. 18180748 10.1038/ismej.2007.95

[73] DeVries EJ, Breeuwer JAJ, Jacobs G, Mollema C (2001) The association of western flower thrips, *Frankliniella occidentalis*, with a near *Erwinia* species gut bacteria: transient or permanent? J Invertebr Pathol 77: 120–128. 11273692 10.1006/jipa.2001.5009

[74] Tóth EM, Hell E, Kovács G, Borsodi AK, Márialigeti K (2006) Bacteria isolated from the different developmental stages and larval organs of the obligate parasitic fly, *Wohlfahrtia magnifica* (Diptera: Sarcophagidae). Microb Ecol 51: 13–21. 16382282 10.1007/s00248-005-0090-6

[75] Panikkar P (2002) Food intake, growth and conversion efficiency in *Macrobrachium rosenbergii* (de Man) and *M. lanchesteri* (de Man). Indian J Fish 49: 29–34.

[76] Barros HP de, Valenti WC (2003) Food intake of *Macrobrachium rosenbergii* during larval development. Aquaculture 216: 165–176.

[77] Letunic I, Bork P (2007) Interactive Tree Of Life (iTOL): an online tool for phylogenetic tree display and annotation. Bioinformatics 23: 127–128. 17050570 10.1093/bioinformatics/btl529

