# Supplementary material for: The Impact of Environmental Heterogeneity and Life Stage on the Hindgut Microbiota of Holotrichia parallela Larvae (Coleoptera: Scarabaeidae)
Source: PLoS One. 2013 Feb 21;8(2):e57169. doi: 10.1371/journal.pone.0057169 (PMC3578786; doi:10.1371/journal.pone.0057169)
Supplement: Table S1 — (DOC) [file pone.0057169.s003.doc]

Table S1. ∫-LIBSHUFF *P* values for pairwise comparisons of clone libraries of different natural populations (ΔCXY lower-left triangular, ΔCYX upper-right triangular)

|  | FJ | GD | GX | HB | NX | SC | SD | LN | TJ | ZJ |
| --- | --- | --- | --- | --- | --- | --- | --- | --- | --- | --- |
| FJ |  | 0.0327 | **＜0.0001** | **＜0.0001** | **0.0002** | **0.0013** | **0.0001** | **0.0005** | **0.0007** | 0.0011 |
| GD | **0.0001** |  | **＜0.0001** | **＜0.0001** | **＜0.0001** | **＜0.0001** | **0.0001** | **＜0.0001** | **＜0.0001** | **＜0.0001** |
| GX | **＜0.0001** | **＜0.0001** |  | **＜0.0001** | **＜0.0001** | **＜0.0001** | **＜0.0001** | **＜0.0001** | **＜0.0001** | **＜0.0001** |
| HB | **＜0.0001** | **＜0.0001** | **＜0.0001** |  | **＜0.0001** | **＜0.0001** | **＜0.0001** | **＜0.0001** | **0.0001** | **＜0.0001** |
| NX | **＜0.0001** | **0.0002** | **＜0.0001** | **＜0.0001** |  | 0.0485 | **＜0.0001** | **＜0.0001** | **0.0002** | 0.0023 |
| SC | **＜0.0001** | 0.0014 | **＜0.0001** | **＜0.0001** | **0.0010** |  | **0.0006** | **＜0.0001** | **＜0.0001** | **＜0.0001** |
| SD | **＜0.0001** | 0.0101 | **＜0.0001** | **＜0.0001** | **＜0.0001** | 0.0245 |  | **＜0.0001** | **＜0.0001** | **＜0.0001** |
| LN | **＜0.0001** | **＜0.0001** | **＜0.0001** | **＜0.0001** | **＜0.0001** | **＜0.0001** | **＜0.0001** |  | **＜0.0001** | **＜0.0001** |
| TJ | **＜0.0001** | **＜0.0001** | **＜0.0001** | **＜0.0001** | **＜0.0001** | **＜0.0001** | **＜0.0001** | **＜0.0001** |  | **＜0.0001** |
| ZJ | 0.0308 | 0.0406 | **＜0.0001** | **＜0.0001** | 0.0014 | 0.0019 | **＜0.0001** | **0.0001** | 0.0013 |  |

Boldface values indicate signiﬁcant *P* values (*P*<0.0011) after Bonferroni correction for multiple pairwise comparisons. Libraries are distinct from one another if either of the comparisons (X versus Y or Y versus X) is signiﬁcant.
