# Supplementary material for: The Impact of Environmental Heterogeneity and Life Stage on the Hindgut Microbiota of Holotrichia parallela Larvae (Coleoptera: Scarabaeidae)
Source: PLoS One. 2013 Feb 21;8(2):e57169. doi: 10.1371/journal.pone.0057169 (PMC3578786; doi:10.1371/journal.pone.0057169)
Supplement: Table S2 — (DOC) [file pone.0057169.s004.doc]

Table 4 ∫-LIBSHUFF *P* values for pairwise comparisons of clone libraries of three instars (ΔCXY lower-left triangular, ΔCYX upper-right triangular)

|  | 1st | 2nd | 3rd |
| --- | --- | --- | --- |
| 1st |  | **＜0.0001** | **0.004** |
| 2nd | **0.0043** |  | 0.0406 |
| 3rd | **0.0019** | 0.3647 |  |

Boldface values indicate signiﬁcant *P* values (*P*<0.0083). The consensus for ∫-LIBSHUFF analyses is that if either XY or YX has a *P* value of <0.0083, the two communities are significantly different.
